# Pyoluteorin and 2,4-diacetylphloroglucinol are major contributors to *Pseudomonas protegens* Pf-5 biocontrol against *Botrytis cinerea* in cannabis

**DOI:** 10.3389/fmicb.2022.945498

**Published:** 2022-08-09

**Authors:** Carole Balthazar, Renée St-Onge, Geneviève Léger, Simon G. Lamarre, David L. Joly, Martin Filion

**Affiliations:** ^1^Department of Biology, Université de Moncton, Moncton, NB, Canada; ^2^Agriculture and Agri-Food Canada, Saint-Jean-sur-Richelieu Research and Development Center, Saint-Jean-sur-Richelieu, QC, Canada

**Keywords:** *Botrytis cinerea*, gray mold, *Cannabis sativa*, *Pseudomonas protegens*, antibiosis, pyoluteorin, 2,4-diacetylphloroglucinol, deletion mutant

## Abstract

*Pseudomonas protegens* Pf-5 is an effective biocontrol agent that protects many crops against pathogens, including the fungal pathogen *Botrytis cinerea* causing gray mold disease in *Cannabis sativa* crops. Previous studies have demonstrated the important role of antibiotics pyoluteorin (PLT) and 2,4-diacetylphloroglucinol (DAPG) in Pf-5-mediated biocontrol. To assess the potential involvement of PLT and DAPG in the biocontrol exerted by Pf-5 against *B. cinerea* in the phyllosphere of *C. sativa*, two knockout Pf-5 mutants were generated by in-frame deletion of genes *pltD* or *phlA*, required for the synthesis of PLT or DAPG respectively, using a two-step allelic exchange method. Additionally, two complemented mutants were constructed by introducing a multicopy plasmid carrying the deleted gene into each deletion mutant. *In vitro* confrontation assays revealed that deletion mutant ∆*pltD* inhibited *B. cinerea* growth significantly less than wild-type Pf-5, supporting antifungal activity of PLT. However, deletion mutant ∆*phlA* inhibited mycelial growth significantly more than the wild-type, hypothetically due to a co-regulation of PLT and DAPG biosynthesis pathways. Both complemented mutants recovered *in vitro* inhibition levels similar to that of the wild-type. In subsequent growth chamber inoculation trials, characterization of gray mold disease symptoms on infected cannabis plants revealed that both ∆*pltD* and ∆*phlA* significantly lost a part of their biocontrol capabilities, achieving only 10 and 19% disease reduction respectively, compared to 40% achieved by inoculation with the wild-type. Finally, both complemented mutants recovered biocontrol capabilities *in planta* similar to that of the wild-type. These results indicate that intact biosynthesis pathways for production of PLT and DAPG are required for the optimal antagonistic activity of *P. protegens* Pf-5 against *B. cinerea* in the cannabis phyllosphere.

## Introduction

*Pseudomonas* is a large genus of ubiquitous Gammaproteobacteria that has received much attention for the development of biocontrol agents over the years. These versatile bacteria are indeed well-known for their great metabolic flexibility and lifestyle adaptability, allowing them to colonize a wide range of environmental niches, including plant roots and their associated soil (rhizosphere), and plant aerial surfaces (phyllosphere; Gross and Loper, 2009). The model strain *Pseudomonas protegens* Pf-5 was first isolated from cotton seedling rhizosphere in Texas, United States (Howell and Stipanovic, 1979) and has since been used to control the growth and/or development of various plant pathogens in cotton, cucumber, pea, maize, wheat, turfgrass, tomato, and potato crops (Loper et al., 2007). The primary mode of action of this successful antagonistic strain relies on the production of antimicrobial metabolites with wide-spectrum antifungal activities, including hydrogen cyanide (HCN), pyrrolnitrin (PRN), pyoluteorin (PLT), 2,4-diacetylphloroglucinol (DAPG), orfamides, and rhizoxins (Fernando et al., 2005; Gross and Loper, 2009).

The polyketide DAPG is a phenolic compound synthesized by acetylation of its precursor monoacetylphloroglucinol (MAPG), itself produced *via* a phloroglucinol intermediate formed by condensation of three malonyl-CoA molecules. The genes required for DAPG biosynthesis are located within a highly conserved 6.5-kb genomic DNA fragment comprising nine open reading frames (*phlACBDEFGHI*) grouped together in a biosynthetic gene cluster. Four of these genes (*phlACBD*) constitute the operon directly involved in the biosynthesis, while the others (*phlEFGHI*) code for efflux, degradation, and regulatory proteins (Gross and Loper, 2009; Biessy and Filion, 2021). Notably, *phlA*, *phlC*, and *phlB* encode the subunits of a multimeric enzyme that catalyzes the conversion of phloroglucinol to MAPG, and of MAPG to DAPG (Pavkov-Keller et al., 2019). The antibiotic DAPG, produced by strain Pf-5 and other related *Pseudomonas* strains, has been found effective against fungal pathogens such as *Rhizoctonia solani* (Nowak-Thompson et al., 1994), *Gaeumannomyces tritici*, *Thielaviopsis basicola* (Vincent et al., 1991; Keel et al., 1992; Kwak et al., 2009), *Fusarium verticillioides*, *Fusarium oxysporum* (Sharifi-Tehrani et al., 1998; Quecine et al., 2016), *Monilinia fructicola*, and *Botrytis cinerea* (Zhang et al., 2020), as well as against the oomycetes *Pythium ultimum* (Fenton et al., 1992; Shanahan et al., 1992; Nowak-Thompson et al., 1994; Sharifi-Tehrani et al., 1998; de Souza et al., 2003), *Plasmopara viticola* and *Aphanomyces cochlioides* (Islam and von Tiedemann, 2011), and against various phytopathogenic bacteria (Nowak-Thompson et al., 1994; Cronin et al., 1997a) and nematodes (Cronin et al., 1997b; Meyer et al., 2009). Impairment of mitochondrial functions and calcium homeostasis are the primary mechanisms responsible for the direct toxicity of DAPG against filamentous fungi (Troppens et al., 2013), while induction of systemic immune responses in *Arabidopsis thaliana* has also been reported (Iavicoli et al., 2003; Weller et al., 2012; Chae et al., 2020).

On the other hand, the antibiotic PLT is composed of a dichlorinated pyrrole moiety and a resorcinol ring that are synthesized by a polyketide synthase-non-ribosomal peptide synthetase hybrid pathway. The biosynthetic gene cluster encompasses 17 genes involved in PLT production (*pltABCDEFGLM*), regulation (*pltZ and pltR*), and efflux (*pltIJKNOP*), spanning ~30 kb of DNA in the genome of *P. protegens* Pf-5 (Gross and Loper, 2009). Notably, *pltD* is part of the main structural operon and encodes a putative halogenase which plays an essential and rate-limiting role in the production of PLT, even though its exact function remains unclear (Nowak-Thompson et al., 1999; Li et al., 2012b; Zhang et al., 2020). While numerous antifungal and antibacterial effects have been reported, PLT produced by strain Pf-5 is best known for its inhibitory activity against the oomycete *P. ultimum* (Howell and Stipanovic, 1980; Maurhofer et al., 1994; Gross and Loper, 2009; Clifford et al., 2016).

Cannabis plants (*Cannabis sativa*) have been cultivated worldwide for centuries to produce fiber and oilseeds (commonly referred to as hemp crops), as well as medicinal and recreational compounds (commonly referred to as marijuana crops). With the ongoing easing of cannabis prohibition laws in several countries like Canada, a renewed interest in large-scale cultivation is accompanied by the emergence of plant pathogens impacting cannabis yield and harvest quality (Punja, 2021). Among the pathogens of greatest concern for both hemp and marijuana crops is *B. cinerea*, the causal agent of bud rot and gray mold disease which are responsible for devastating damages in outdoor and indoor cannabis cropping systems (McPartland et al., 2000; Punja and Ni, 2021). Recent reviews exploring the potential benefits of biocontrol agents for cannabis crops have highlighted clear opportunities regarding the inoculation of beneficial *Pseudomonas* spp. to control *B. cinerea* in cannabis crops, including *P. protegens* Pf-5 (Lyu et al., 2019; Taghinasab and Jabaji, 2020; Balthazar et al., 2022a,b), even though supporting validation studies providing mechanistic insights are still largely lacking. Therefore, the aim of this study was to investigate the contribution of the antibiotics PLT and DAPG in the biocontrol exerted by *P. protegens* Pf-5 against the fungal pathogen *B. cinerea* infecting *C. sativa* plants. It was previously shown that *P. protegens* Pf-5 was able to significantly reduce gray mold symptom severity on *C. sativa* leaves when applied before pathogen infection (Balthazar et al., 2022b). Here, we report that this biocontrol protection is significantly impaired in Pf-5 isogenic knockout mutants where genes required for PLT or DAPG biosynthesis have been deleted. Antibiotics PLT and DAPG are thus proposed as key determinants of *P. protegens* Pf-5 biocontrol success against *B. cinerea* within the cannabis phyllosphere.

## Materials and methods

### Bacterial growth conditions

*Escherichia coli* and *Pseudomonas protegens* strains (Table 1) were routinely grown at 37 and 25°C, respectively, in Lennox’s lysogeny broth (LB; 10 g L^−1^ peptone or tryptone, 5 g L^−1^ yeast extract, and 5 g L^−1^ NaCl) and on Lennox’s LB agar (Lennox’s LB supplemented with 12 g L^−1^ agar; modified from Lennox, 1955). When appropriate, the medium was supplemented with antibiotics: 100 μg ml^−1^ ampicillin sodium salt, 25 μg ml^−1^ chloramphenicol, 15 μg ml^−1^ (*E. coli*), or 30 μg ml^−1^ (*P. protegens*) gentamicin sulfate. All plate cultures were prepared in 100-mm-diameter Petri dishes.

**Table 1:** Bacterial strains and plasmids.

Strain/plasmid	Genotype, properties, and/or uses^a^	Source
*P. protegens* strains		
Pf-5	Biocontrol strain; cotton seedling rhizosphere isolate producing 2,4-diacetylphloroglucinol and pyoluteorin; Amp^R^ Chl^R^ Spt^R^ Str^R^ Tet^R^	Howell and Stipanovic, 1979
Pf-5∆*phlA*	Pf-5∆*phlA* (∆*PFL_5954*; markerless deletion of +4 to +1,080)	This study
Pf-5∆*pltD*	Pf-5∆*pltD* (∆*PFL_2790*; markerless deletion of +4 to +1,620)	This study
*E. coli strains*		
DH5α	Plasmid construction and storage strain; F-ϕ80dlacZ∆M15 ∆(*lacIZYA-argF*)*U169 recA1 endA1 hsdR17*(*rK^−^*, *mK^+^*) *deoR supE44 thi-1 gyrA96 relA1*; Nal^R^	BioPioneer
Plasmids		
pEX18Gm	Mobilizable (but not self-transmissible) suicide vector; *ori* (pMB1, high-copy mutant) *oriT sacB lacZα*; Gen^R^	Hoang et al., 1998
pEX18Gm-∆*phlA*	Allelic exchange vector; *phlA* (*PFL_5954*) upstream flanking DNA (−2,343 to +3) fused to *phlA* downstream flanking DNA (+1,081 to +3,459), directionally cloned in the *Hin*dIII-*Kpn*I site of pEX18Gm	This study
pEX18Gm-∆*pltD*	Allelic exchange vector; *pltD* (*PFL_2790*) upstream flanking DNA (−2,330 to +3) fused to *pltD* downstream flanking DNA (+1,621 to +3,876), directionally cloned in the *Hin*dIII-*Kpn*I site of pEX18Gm	This study
pRK600	Self-transmissible helper plasmid; *ori* (ColE1) *oriT tra*; Chl^R^	Finan et al., 1986
pUCP22	*E. coli*-*Pseudomonas* shuttle vector; *ori* (pMB1, high-copy mutant) *ori* (pRO1600) *rep lacZα*; Amp^R^ Gen^R^	West et al., 1994
pUCP22-*phlA*	Mutant complementation vector; *phlA* (*PFL_5954*) (−60 to +1,099), directionally cloned in the *Bam*HI-*Kpn*I site under the transcriptional control of P*_lac_*	This study
pUCP22-*pltD*	Mutant complementation vector; *pltD* (*PFL_2790*) (−69 to +1,642) with an additional stop codon inserted at its 3′ end, directionally cloned in the *Bam*HI-*Kpn*I site under the transcriptional control of P*_lac_*	This study

a Base numbering is relative to the start codon.

Amp^R^, ampicillin-resistant; Chl^R^, chloramphenicol-resistant; Gen^R^, gentamicin-resistant; Nal^R^, nalidixic acid-resistant; Spt^R^, spectinomycin-resistant; Str^R^, streptomycin-resistant; Tet^R^, tetracycline-resistant; and P*_lac_*, *lac* promoter.

### DNA extractions

*Pseudomonas protegens* genomic DNA was isolated from 24-h-old broth-grown cells using the DNeasy UltraClean Microbial kit (Qiagen, Toronto, ON, Canada). The manufacturer’s protocol was followed, with the exception that cell lysis was carried out using a FastPrep-24 (MP Biomedicals, Solon, OH, United States; 4 m s^−1^ for 30 s) instead of a vortex. Plasmid DNA was extracted from 18-to 20-h-old broth-grown *E. coli* cells using standard procedures (Sambrook and Russell, 2001). DNA was quantified by spectrophotometry, and its integrity was confirmed by agarose gel electrophoresis.

### Construction of allelic exchange vectors

**Figure 1:**
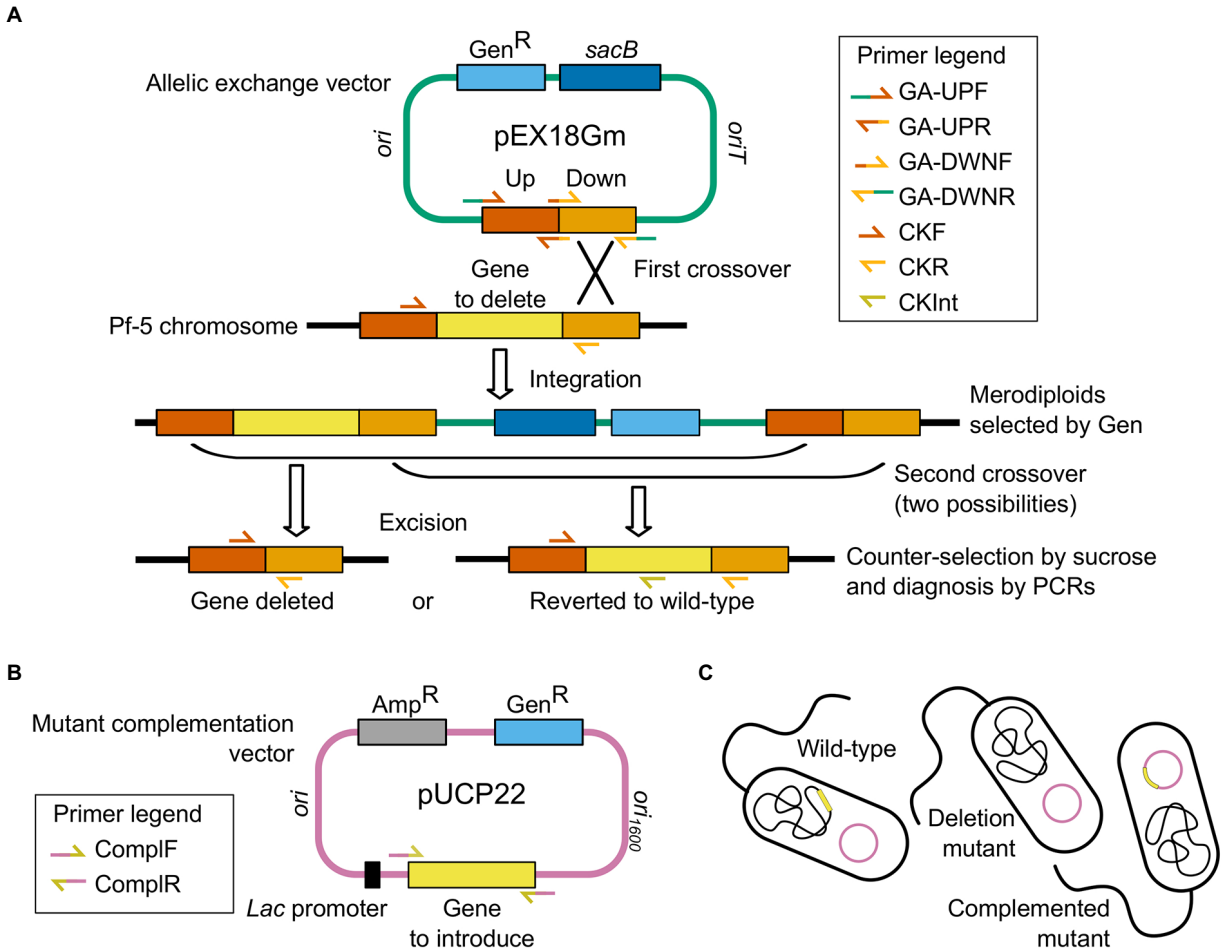
Generation of *Pseudomonas protegens* Pf-5 knockout mutants and complemented mutants by two-step allelic exchange and plasmid-based complementation. **(A)** Construction of the allelic exchange vector for in-frame deletion of *phlA* or *pltD* (yellow) in the bacterial wild-type genome. The gene knockout cassette comprises the DNA sequences immediately upstream and downstream of the gene to be deleted (Up and Down flanks, red and orange) and is assembled into the suicide vector pEX18Gm (green) carrying the gentamicin resistance (Gen^R^, light blue) and sucrose sensitivity (*sacB*, dark blue) genes. Integration of the allelic exchange vector in Pf-5 chromosome occurs by homologous recombination (first crossover) after bacterial conjugation, and merodiploids are selected on gentamicin-amended media. Subsequent homologous recombination (second crossover) results in the loss of the allelic exchange vector backbone, which is selected with sucrose-amended media. Depending on the second crossover locus, vector excision either restores the wild-type allele or deletes the gene in the bacterial chromosome. Clones with the correct genotype (gene deleted) are discriminated from reverted clones by diagnostic PCRs. **(B)** Construction of the complementation vector for electroporation into complemented mutants, resulting in plasmid-based complementation of *phlA* or *pltD* (yellow). The gene (yellow) is assembled into the multicopy shuttle vector pUCP22 (pink), under transcriptional control of a constitutive *lac* promoter (black), which carries the gentamicin resistance (Gen^R^, light blue) and ampicillin resistance (Amp^R^, gray) genes. **(C)** The empty complementation vector (pink plasmid) is introduced into the wild-type bacteria (undisturbed chromosome in black with target gene in yellow) and the deletion mutants (knockout chromosome in black missing target gene), while the recombinant complementation vector (pink plasmid carrying target gene in yellow) is introduced into the complemented mutants (knockout chromosome in black missing target gene). Combined steps thus result in unscarred mutants that differ from the wild-type strain only in the presence or absence of the targeted gene (yellow). Primers used to monitor each key step are indicated in the corresponding legends. Drawings are not to scale.

Genes *phlA* (+4 to +1,080) and *pltD* (+4 to +1,620; numbering relative to the gene’s start codon) were deleted in-frame from the *P. protegens* Pf-5 genome (GenBank accession no. CP000076; Paulsen et al., 2005) using a two-step allelic exchange strategy based on (Hmelo et al., 2015; Figure 1). To avoid polar effects, the target gene’s start and stop codons were not deleted, and care was taken to retain the downstream encoded gene’s putative ribosome-binding site and start codon, particularly when translational coupling was suspected (Supplementary Figures S1, S2).

For each gene to be deleted, a gene knockout cassette, comprising a mutant allele bordered on either side by DNA sequences flanking the region of the *P. protegens* chromosome to be deleted, was synthesized and assembled into an allelic exchange vector, as follows. To construct each knockout cassette, DNA sequences located immediately upstream and downstream of the gene to be deleted (hereafter called the upstream and downstream flanks) were individually PCR-amplified from *P. protegens* Pf-5 genomic DNA using Phusion High-Fidelity DNA Polymerase (New England Biolabs, Whitby, ON, Canada) with primer pairs GA-UPF/GA-UPR (for amplification of the upstream flank) and GA-DWNF/GA-DWNR (for amplification of the downstream flank). Each primer comprised a 3′ sequence-specific priming sequence, designed using Primer-BLAST (Ye et al., 2012; Available at https://www.ncbi.nlm.nih.gov/tools/primer-blast/). To introduce regions of overlap between the upstream and downstream flank amplicons, and promote their annealing during cassette assembly, a 10-nt extension, designed using the NEBuilder Assembly Tool version 2.5.3 (New England Biolabs; Available at https://nebuilder.neb.com/#!/), was added to the 5′ end of the GA-UPR and GA-DWNF primers. A 20-nt extension was also incorporated into the 5′ end of the GA-UPF and GA-DWNR primers for subsequent assembly of the cassette with the suicide vector pEX18Gm (GenBank accession no. AF047518; Hoang et al., 1998; Table 1) to generate the allelic exchange vector. Primer pairs used, primer sequences, amplification conditions, and product lengths are provided in Supplementary Tables S1, S2. PCR products were then column-purified using the PureLink PCR Purification kit (Invitrogen, Waltham, MA, United States) with the Binding Buffer B2.

The upstream flank, the downstream flank, and linearized pEX18Gm were then assembled together to form the allelic exchange vector. To generate a linearized vector for DNA assembly, plasmid pEX18Gm was digested with FastDigest *Kpn*I (Thermo Scientific, Waltham, MA, United States), column-purified, and then digested with FastDigest *Hin*dIII (Thermo Scientific). The digested plasmid was column-purified, dephosphorylated for 2 h using Quick CIP (New England Biolabs) with approximately twice the recommended mass of DNA, and column-purified again. Then, 13.6 fmol upstream flank, 13.6 fmol downstream flank, and 7.0 fmol dephosphorylated, digested pEX18Gm were assembled together at 50°C for 1 h in 1 × NEBuilder HiFi DNA Assembly Master Mix (New England Biolabs).

The DNA assembly reaction mixture, containing the newly assembled allelic exchange vector, was electroporated into *E. coli* DH5α, as described below. Gentamicin-resistant electrotransformants were then screened for the acquisition of a correctly assembled allelic exchange vector by PCR-amplifying the gene knockout cassette using the DreamTaq Hot Start Green PCR Master Mix (Thermo Scientific) with the CKF/CKR primer pair and crude cell lysates as template (Supplementary Tables S1, S2).

### Triparental mating, and selection of merodiploids and deletion mutants

Each recombinant pEX18Gm plasmid (Table 1) was transferred to *P. protegens* Pf-5 using triparental mating. Briefly, 20–24-h-old starter cultures of the donor (*E. coli* DH5α carrying an allelic exchange vector), helper (*E. coli* DH5α/pRK600), and recipient (*P. protegens* Pf-5) strains (Table 1) were each diluted 50–100-fold in 50 ml Lennox’s LB in a 250-ml Erlenmeyer flask. The medium was supplemented, as needed, with an antibiotic to maintain plasmid selection. Cultures were grown at 37°C (*E. coli*) or 25°C (*P. protegens*) with constant shaking at 250–300 rpm until they reached an optical density of 0.4–0.7 at 600 nm, and cells were collected and washed with glycerol (10% v/v) as described below for the preparation of electrocompetent cells. The final washed cell pellet was then resuspended in 300 μl ice-cold Lennox’s LB, and 100 μl each donor, helper and recipient cell suspension were mixed together and spread-plated on 30 ml Lennox’s LB agar supplemented with 10 mM MgSO_4_. The plate culture was incubated at 28°C for 20 h.

To select for *P. protegens* merodiploids, cells were scraped off the plate’s surface using an inoculation loop, resuspended in 1 ml phosphate-buffered saline (PBS) solution (1×; Sambrook and Russell, 2001), and 5 μl suspension were spread-plated onto 30 ml Lennox’s LB agar supplemented with ampicillin sodium salt (which selects against the ampicillin-sensitive *E. coli* donor and helper strains) and gentamicin sulfate (which selects for *P. protegens* cells that have incorporated the allelic exchange vector into their chromosome by homologous recombination). The plate culture was incubated at 25°C for ~2 days.

*Pseudomonas protegens* clones that have lost the pEX18Gm backbone owing to a second crossover event were then isolated using sucrose-mediated counterselection. Briefly, gentamicin-resistant merodiploid colonies were patched onto a fresh plate of Lennox’s LB agar supplemented with gentamicin sulfate. After ~2 days at 25°C, patches were scraped off the plate using an inoculation loop, resuspended in 1 ml PBS solution (1×), and 10 μl cell suspension were spread-plated onto 30 ml no-salt LB agar (10 g L^−1^ tryptone, 5 g L^−1^ yeast extract, and 15 g L^−1^ agar) supplemented with 10% w/v sucrose (modified from Hmelo et al., 2015). Plate cultures were incubated at 28°C for 22 h. Putative deletion mutants (gentamicin-sensitive) were identified by patching sucrose-resistant clones on 30 ml Lennox’s LB agar supplemented with or without gentamicin sulfate (25°C for 22 h).

Sucrose-resistant, gentamicin-sensitive clones were then screened for the deletion of the target gene by PCR-amplifying the gene knockout cassette using the DreamTaq Hot Start Green PCR Master Mix with the CKF/CKR primer pair and crude cell lysates as template (Supplementary Tables S1, S2). Genomic DNA was isolated from deletion mutants, and successful deletion of the target gene was confirmed by a series of diagnostic PCRs targeting the wild-type (primer pair CKF/CKInt) and mutant (primer pair CKF/CKR) alleles (Supplementary Tables S1, S2). Genotype and phenotype confirmation results are provided in Supplementary Figures S1, S2.

### Mutant complementation

To complement the mutations, the deleted gene’s coding region—with 60–69 bp of upstream sequence encompassing the putative ribosome-binding site—was placed under the transcriptional control of a constitutive *lac* promoter and reintroduced into the deletion mutant on the multicopy shuttle vector pUCP22 (GenBank accession no. U07166; West et al., 1994; Table 1). Care was taken to avoid including the downstream gene’s ribosome-binding site and start codon. When the inclusion of these features was unavoidable, additional codons of the downstream gene were included in the complementation construct, followed by an in-frame opal stop codon (Supplementary Figure S3).

To construct the complementation vectors, *phlA* (−60 to +1,099) and *pltD* (−69 to +1,642; numbering relative to the start codon) were first PCR-amplified from *P. protegens* Pf-5 genomic DNA using Phusion High-Fidelity DNA Polymerase with the ComplF/ComplR primer pair (Supplementary Tables S1, S2). Different restriction sites were engineered into the 5′ end of each primer to enable subsequent restriction cloning of the PCR product into pUCP22 (Supplementary Table S1). PCR products were column-purified as above.

Products were then sequentially digested, first with FastDigest *Kpn*I, and then with *Bam*HI (Thermo Scientific). Products were column-purified after each digestion. Plasmid pUCP22 was similarly digested, dephosphorylated and purified as described for pEX18Gm, and then ligated with purified digested PCR product for 18 h at 16°C using 0.1 U μl^−1^ T4 DNA Ligase (Invitrogen), thereby cloning the PCR product immediately downstream of the vector’s *lac* promoter. After heat-inactivating the enzyme at 65°C for 20 min, the ligase reaction mixtures, containing the newly constructed complementation plasmids, were electroporated into electrocompetent *E. coli* DH5α as described below.

Gentamicin-resistant *E. coli* electrotransformants were screened for the presence of a recombinant pUCP22 plasmid (Table 1) by PCR-amplifying the insert using the DreamTaq Hot Start Green PCR Master Mix with the ComplF/ComplR primer pair and crude cell lysates as template (Supplementary Tables S1, S2). Plasmids were then isolated from positive electrotransformants.

Each confirmed recombinant plasmid was then electroporated into its corresponding *P. protegens* deletion mutant as described below. The empty plasmid was also electroporated into the wild-type *P. protegens* Pf-5 and each deletion mutant. To confirm the successful introduction of each complementation construct into its corresponding deletion mutant, each plasmid’s insert was PCR-amplified using the DreamTaq Hot Start Green PCR Master Mix with the appropriate ComplF/ComplR primer pair and crude cell lysate as template (Supplementary Tables S1, S2). Genotype confirmation results are provided in Supplementary Figure S3.

### Electroporation

Electroporations of *E. coli* and *P. protegens* were carried out following a protocol adapted from (Gust et al., 2003). Electrocompetent cells were first prepared by washing exponential-phase cells with glycerol (10% v/v) as follows. An 18-h-old starter culture of the recipient was diluted 50–100-fold in 50 ml modified super optimal broth (20 g L^−1^ tryptone, 5 g L^−1^ yeast extract, and 0.5 g L^−1^ NaCl; adapted from Sambrook and Russell, 2001) in a 250-ml Erlenmeyer flask, and the culture was incubated at 37°C (*E. coli*) or 25°C (*P. protegens*) with constant shaking at 250–300 rpm until the culture reached an optical density of 0.3–0.5 at 600 nm. Then, 42–45 ml culture were transferred to a 50-ml centrifuge tube and cooled on ice for at least 5 min. Cells were pelleted by centrifugation at 3,950 × *g* for 10 min at 4°C, and the culture supernatant was decanted and discarded. The pelleted cells were resuspended in 42–45 ml ice-cold glycerol (10% v/v) and pelleted once more as above. The supernatant was decanted, and cells were washed a second time with 5 ml ice-cold glycerol (10% v/v) and pelleted. After decanting the supernatant, the pelleted cells were resuspended in the small volume of supernatant remaining in the centrifuge tube. Then, 50 μl washed cells were mixed with either 1 μl DNA assembly reaction mixture, 3 μl ligation reaction mixture, or 1 μl plasmid extract in a cold 1.5-ml microcentrifuge tube, and the suspension was subsequently transferred to a pre-chilled 2-mm-gapped Gene Pulser Cuvette (Bio-Rad, Mississauga, ON, Canada). The electroporation was carried out in a Gene Pulser Xcell Electroporation System (Bio-Rad) using an exponential decay pulse (25 μF, 2.5 kV, and 200 Ω). Shocked cells were immediately resuspended in 1 ml ice-cold Lennox’s LB, transferred to a new chilled 1.5-ml microcentrifuge tube, and incubated at 37°C for 1 h (*E. coli*) or 25°C for 2 h (*P. protegens*) with constant shaking at 250–300 rpm. Culture aliquots were then spread-plated on 30 ml Lennox’s LB agar supplemented with gentamicin sulfate to select for electrotransformants. Plate cultures were incubated at 37°C for ~1 day (*E. coli*) or 25°C for ~2 days (*P. protegens*).

### Fungal growth inhibition *in vitro*

The ability of *P. protegens* Pf-5 and its derivatives to inhibit the growth of *B. cinerea in vitro* was assessed using confrontation assays described previously (Balthazar et al., 2022b). A pathogenic strain of *B. cinerea*, isolated from symptomatic *C. sativa* plants in British Columbia, Canada (Punja et al., 2019), was kindly provided by Z.K. Punja (Simon Fraser University, BC, Canada). Actively growing cultures were routinely maintained at 25°C on potato dextrose agar (PDA; Difco, BD, Franklin Lakes, NJ, United States), and mycelial plugs (5 mm in diameter) were harvested from the edge of the colony. Bacterial cells were collected from Lennox’s LB-gentamicin broth cultures no older than 24 h, washed twice with sterile PBS solution (1×) with centrifugation at 3,950 × *g* for 5 min at 4°C, then resuspended to a final concentration of 10^8^ CFU ml^−1^ in PBS solution (1×), using standard curves and optical density readings at 600 nm. Two 10-μl drops of each normalized bacterial suspension were spotted at equal distance (30 mm) from a mycelium plug placed in the center of a Petri plate containing 20 ml fresh PDA medium. Drops containing sterile PBS solution (1×) were added in control Petri plates. Four Petri plates were prepared for each bacterial strain, and the experiment was replicated a second time. Plate cultures were incubated at 25°C in the dark until the mycelium reached the edges of the control plates (~5 days). Absence of mycelial growth around the bacterial colonies in the treated plates reflected their ability to inhibit *B. cinerea* growth, and the corresponding inhibition zones were measured.

### Cannabis gray mold disease reduction assays

The biocontrol ability of *P. protegens* Pf-5 and its derivatives to reduce gray mold severity on cannabis plants was assessed using growth chamber trials as described previously (Balthazar et al., 2022b). Briefly, seeds of *C. sativa* cultivar “Anka” (hemp type) obtained from Céréla (Saint-Hugues, QC, Canada) were germinated in peat-based potting mix (75% v/v Pro-Mix, 25% v/v vermiculite; Premier Tech, Rivière-du-Loup, QC, Canada) at 23°C, 70% relative humidity, 300 μmol m^−2^ s^−1^ light intensity, and 18/6 h (light/dark) photoperiod in a PGR15 growth chamber (Conviron, Winnipeg, MB, Canada). After 7 days, cannabis seedlings were transplanted into individual 4-in diameter pots and grown under the same conditions as above. Plants were inoculated with bacteria after 5 additional days. Bacterial cells were collected from broth cultures no older than 24 h, washed twice in PBS solution (1×) as described above, and resuspended to a final concentration of 10^5^ CFU ml^−1^ in water, as previously described (Balthazar et al., 2022b). The aerial parts of each plant were sprayed with ~10 ml normalized bacterial suspension (treated plants), or an equivalent amount of water (control plants). Each treatment included 12 independent plants arranged randomly in the growth chamber. Plants were infected with *B. cinerea* conidia 2 days later. Conidia were harvested in water from PDA cultures that had been incubated under light beforehand to induce fungal sporulation. Conidia concentration was measured with a hemocytometer and diluted to 10^3^ conidia ml^−1^ in a solution of 0.067 M KH_2_PO_4_ and 0.11 M glucose to promote infection (Van Den Heuvel, 1981). Two 10-μl drops of normalized conidia suspension were spotted onto one leaf per plant (on each side of the central vein of the main leaflet from the second true leaf pair), and plants were kept under high humidity in clear plastic bags until control plants without bacteria exhibited strong disease symptoms (~8 days post infection). Disease severity was evaluated according to an ordinal scoring scale with the following symptom classes: 0, no symptoms; 1, chlorosis without lesions; 2, localized lesions; and 3, spreading and/or sporulating necrotic lesions (Balthazar et al., 2022b). The experiment was replicated a second time, totaling 24 plants per treatment. The disease severity index (DSI) for each treatment was calculated as


$$DSI=\overset{24}{\underset{n=1}{\sum }}\left(score_{n}\right)÷\left(3\times 24\right)\times 100\%$$

which corresponds to the sum of each score obtained by the 24 plants, divided by the highest possible score on the scale and the total number of plants, in percentage. The disease reduction index (DRI) for each treatment was then calculated as


$$DRI=\left(DSI_{control}-DSI_{treated}\right)÷DSI_{control}\times 100\%$$

which corresponds to the reduction of disease severity achieved by each bacterial treatment compared to the control treatment without bacteria, relative to the disease severity of the control treatment, in percentage.

### Statistical analyses

Kruskal Wallis rank sum tests were performed to examine the effect of the bacterial treatments on the inhibition zones in Petri plates and on the count of cannabis leaves in symptom classes. If the test was significant at the 0.05 confidence level, Fisher’s Least Significant Difference (LSD) *post hoc* comparisons with Benjamini-Hochberg correction were carried out to identify which bacterial mutants differed from the wild-type group (R version 4.1.0, package agricolae).

## Results

### PLT and DAPG contribute significantly to fungal growth inhibition *in vitro*

**Figure 2:**
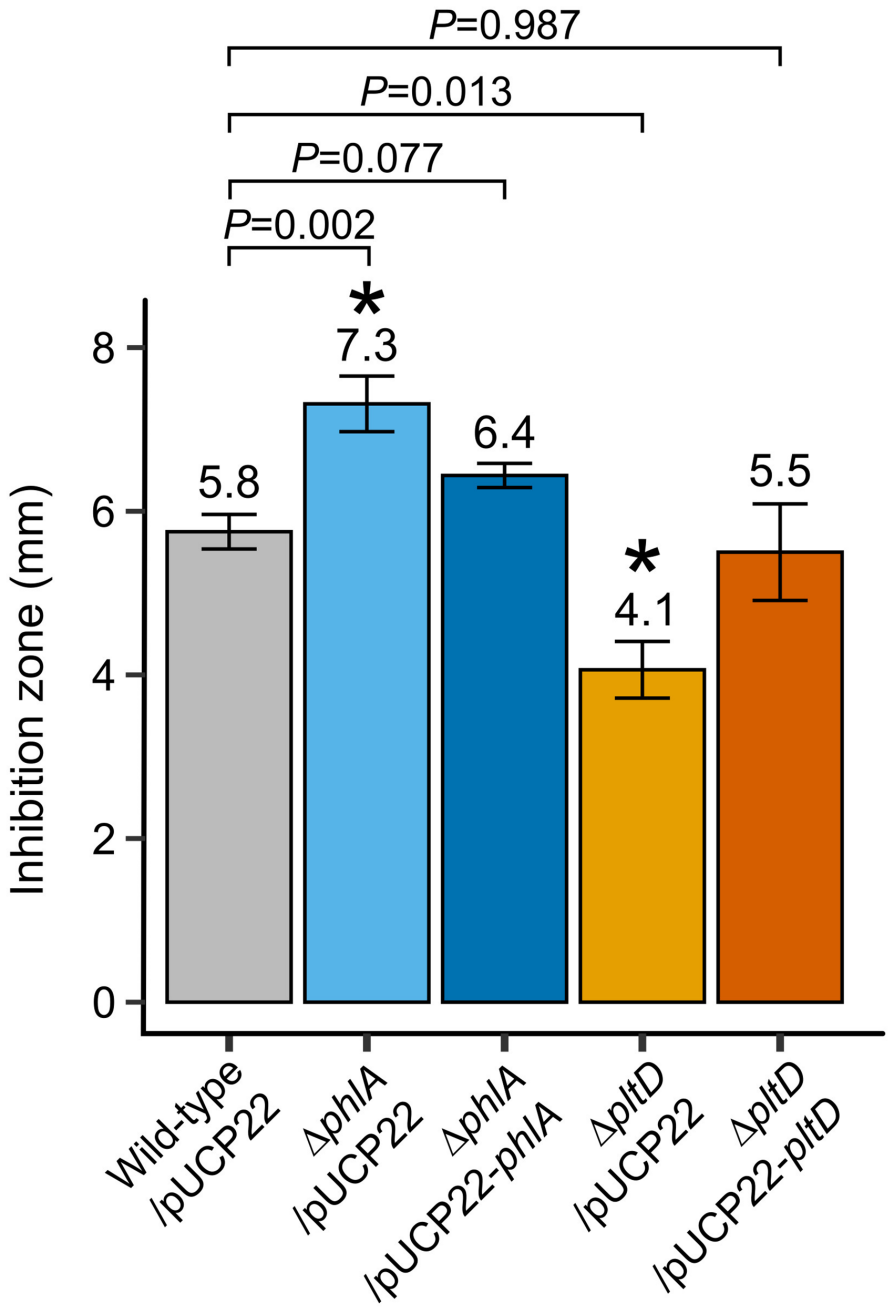
*In vitro* confrontation assays of *Pseudomonas protegens* Pf-5-derived strains against *Botrytis cinerea* mycelium growth. Potato dextrose agar plates containing plugs of *B. cinerea* mycelium and drops of normalized bacterial suspensions (10^8^ CFU ml^−1^ in PBS solution) were incubated at 25°C in the dark. Inhibition zones between the bacterial colonies and mycelium growth were measured after 5 days. Means (indicated above each bar) and standard errors are from two independent experiments containing four replicates each (*n* = 8). Exact *p* values are indicated for comparisons with wild-type/pUCP22 according to Fisher’s LSD *post-hoc* analysis with Benjamini-Hochberg correction, * represents *p* < 0.05.

*In vitro* confrontation assays with bacterial mutants were used to examine whether production of PLT and DAPG played a significant role in the ability of *P. protegens* Pf-5 to inhibit *B. cinerea* mycelial growth. Compared to wild-type Pf-5/pUCP22, the inhibitory capability of deletion mutant ∆*pltD*/pUCP22 was significantly reduced while, paradoxically, that of deletion mutant ∆*phlA*/pUCP22 was significantly increased (Figure 2). Complementation in both mutants, obtained by reintroducing the corresponding deleted gene under the control of a constitutive *lac* promoter on the pUCP22 vector plasmid, restored their inhibition levels to that of wild-type Pf-5/pUCP22 (Figure 2), indicating that the mutations did not have polar effects. Introduction of the empty plasmid pUCP22 (without insert) in the original wild-type and knockout strains ensured that the only difference between these strains and the complemented strains would be the presence of genes *phlA* or *pltD*, rather than the additional presence of plasmid pUCP22 (which might have otherwise influenced the bacterial phenotypes; Figure 1C).

### PLT and DAPG contribute significantly to gray mold reduction *in planta*

**Figure 3:**
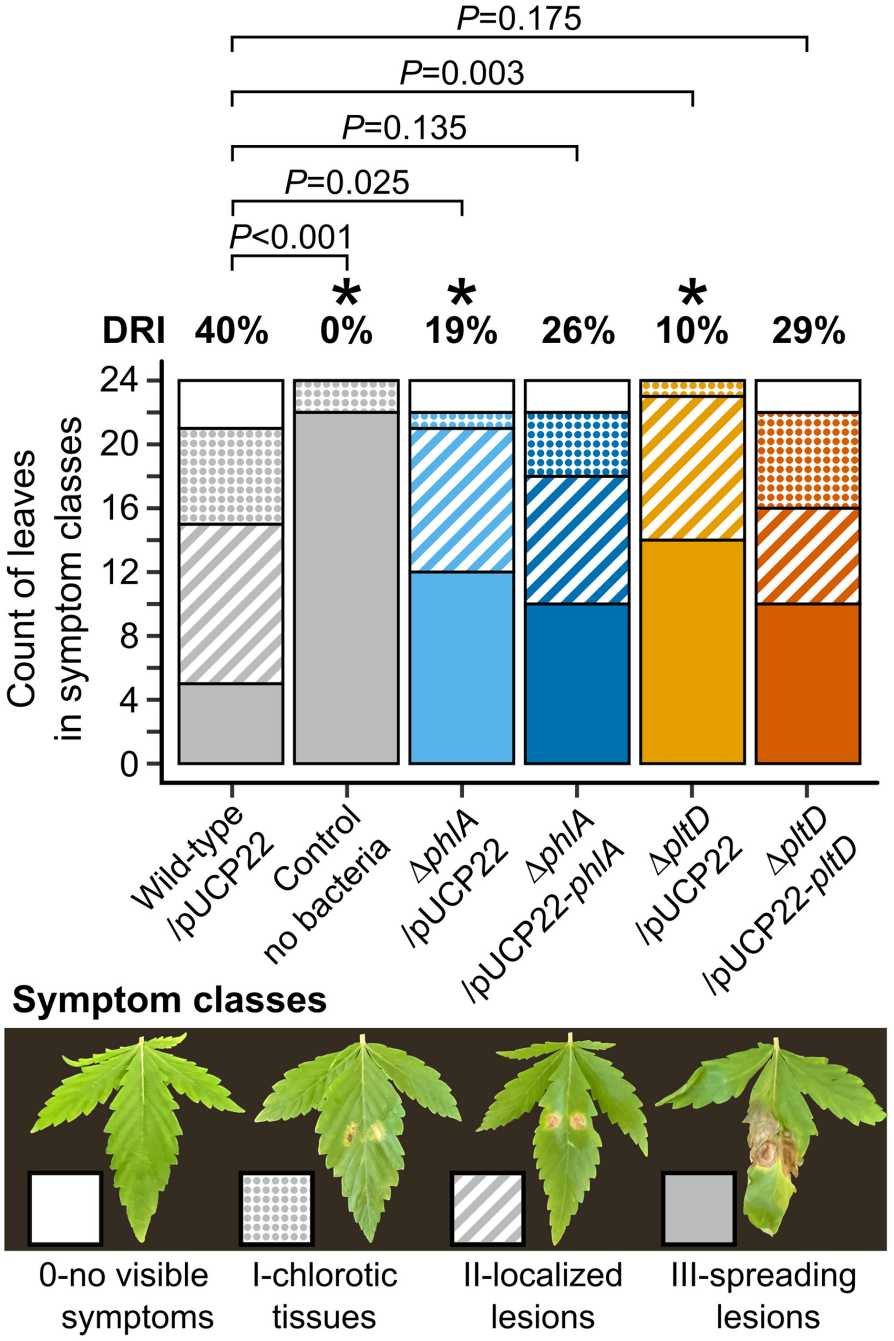
*In planta* disease reduction assays of *P. protegens* Pf-5-derived strains against cannabis gray mold disease. Leaves of 12-day-old cannabis plants were sprayed with normalized bacterial suspensions (10^5^ CFU ml^−1^ in water), or water for control plants. Leaves were infected 2 days later with two drops of *B. cinerea* conidia suspension (10^3^ conidia ml^−1^). Symptoms were observed 8 days after infection when control plants exhibited strong disease symptoms, and disease severity was scored using the four-class scale presented by pictures. The disease reduction index (DRI) was calculated for each treatment relative to the control plants without bacteria, as detailed in Methods. Stacked barplots represent the count of leaves in each symptom class from two independent experiments containing 12 plants each (*n* = 24). Exact *p* values are indicated for comparisons with plants treated with wild-type/pUCP22 according to Fisher’s LSD post-hoc analysis with Benjamini-Hochberg correction, * represents *p* < 0.05.

*In planta* disease reduction assays with bacterial mutants were used to examine whether production of PLT and DAPG played a significant role in the biocontrol exerted by *P. protegens* Pf-5 against *B. cinerea* on cannabis leaves. Confirming its biocontrol abilities, wild-type Pf-5/pUCP22 reduced disease symptoms significantly by 40%, corresponding to approximately one third of the plants displaying no symptoms or small chloroses exempt of lesions, compared to control plants that had not received any bacteria and were largely affected by necrotic lesions (Figure 3). Conversely, the biocontrol capabilities of deletion mutants ∆*phlA*/pUCP22 and ∆*pltD*/pUCP22 were significantly reduced to only 19 and 10% disease reduction, respectively (Figure 3). Complementation in both mutants restored their disease reduction abilities to levels not significantly different from that of wild-type Pf-5/pUCP22, yet slightly inferior (26 and 29%, respectively; Figure 3).

## Discussion

In this study, the potential involvement of the antibiotics PLT and DAPG in the biocontrol exerted by *P. protegens* Pf-5 against *B. cinerea* infecting *C. sativa* plants was investigated with knockout mutants. The two-step double crossover mutagenesis method, previously used for precise in-frame deletions in *Pseudomonas aeruginosa* (Hmelo et al., 2015), was successfully used to generate unscarred unmarked *P. protegens* mutants (Figure 1). To the best of our knowledge, this is the first validation study to establish the importance of specific molecular determinants in a biocontrol agent with cannabis plants. In other crops, the decisive roles of DAPG and PLT in disease suppression by beneficial *Pseudomonas* strains have already been firmly demonstrated with bacterial mutants deficient in their production (Vincent et al., 1991; Fenton et al., 1992; Keel et al., 1992; Shanahan et al., 1992; Maurhofer et al., 1994; Cronin et al., 1997a,b; Rodriguez and Pfender, 1997; Iavicoli et al., 2003; Weller et al., 2012; Quecine et al., 2016; Zhang et al., 2020) and/or with complementation of deficient mutants recovering biocontrol abilities (Vincent et al., 1991; Fenton et al., 1992; Keel et al., 1992; Cronin et al., 1997a,b; Iavicoli et al., 2003; Weller et al., 2012). In this study, two knockout Pf-5 mutants were generated by precise deletion of genes *pltD* or *phlA*, which encode a halogenase or an enzyme subunit required for the synthesis of PLT or DAPG, respectively. Indeed, in *P. protegens* strains, disruption or deletion of gene *pltD* (Nowak-Thompson et al., 1999; Zhang et al., 2020) or *phlA* (Schnider-Keel et al., 2000; Kidarsa et al., 2011; Henkels et al., 2014; Quecine et al., 2016) has been consistently demonstrated to completely abolish the production of PLT or DAPG, respectively, as measured with high-performance liquid chromatography (HPLC). In *P. protegens* CHA0, complementation of a DAPG-deficient mutant with plasmid pME6261 carrying the wild-type *phlA* gene was also shown to fully restore the ability to produce DAPG *in vitro* (Schnider-Keel et al., 2000; Iavicoli et al., 2003). Additionally, enzymatic assays and protein structure determination further demonstrated that expression of *phlA* is required to provide an essential subunit to the multimeric enzyme complex catalyzing DAPG biosynthesis (Pavkov-Keller et al., 2019). Based on these previous studies and rigorous verification by diagnostic PCR amplification (Supplementary Figures S1, S2), the effective deletion of *pltD* or *phlA* genes in the knockout mutants was confidently interpreted as leading to deficient production of the corresponding antibiotic.

During the subsequent *in vitro* confrontation assays, the inhibitory capability of deletion mutant ∆*pltD*/pUCP22 was significantly reduced compared to wild-type Pf-5/pUCP22, as expected, whereas that of deletion mutant ∆*phlA*/pUCP22 was surprisingly increased (Figure 2). The latter observation could hypothetically be due to overproduction of PLT by mutant ∆*phlA*/pUCP22 *in vitro*. Indeed, overproduction of PLT in *Pseudomonas* mutants deficient in DAPG production has already been reported (Schnider-Keel et al., 2000; Zhang et al., 2020), even though it may depend on the growing media, carbon sources, and incubation conditions used, since wild-type levels of PLT production have also been reported (Kidarsa et al., 2011; Henkels et al., 2014; Quecine et al., 2016). The well-known co-regulation between the biosynthesis pathways of PLT and DAPG has been proposed to explain this observation. Indeed, while both PLT and DAPG act as autoinducers of their own production, they repress each other’s production *via* a crosstalk likely mediated by phloroglucinol (Schnider-Keel et al., 2000; Brodhagen et al., 2004; Kidarsa et al., 2011; Li et al., 2012a; Clifford et al., 2016; Yan et al., 2017). Moreover, as regulation of PLT and DAPG biosynthesis in the genus *Pseudomonas* is notoriously complex and involves transcriptional repressors and activators, quorum sensing systems, and/or global translational regulatory networks responding to environmental cues (Li et al., 2012a; Biessy and Filion, 2021), the effect of a single mutation impairing one biosynthesis pathway can be unsurprisingly pleiotropic, thus resulting in the deregulated production of other compounds, as observed in previous studies (Maurhofer et al., 1994; Rodriguez and Pfender, 1997; Schnider-Keel et al., 2000; Zhang et al., 2020).

The biocontrol capabilities of the bacteria were then examined in cannabis plants infected by *B. cinerea*. Inoculation with deletion mutants ∆*pltD*/pUCP22 or ∆*phlA*/pUCP22 resulted in significantly less disease reduction than with the wild-type Pf-5/pUCP22, while plasmid-based complementation restored the biocontrol abilities of the mutants (Figure 3). These results indicate that intact biosynthesis pathways for production of PLT and DAPG are required for the optimal biocontrol protection exerted by *P. protegens* Pf-5 *in planta* against *B. cinerea*. This conclusion is in accordance with previous studies supporting the major role of PLT and DAPG in the biocontrol success of beneficial *Pseudomonas* strains against plant pathogens (Gross and Loper, 2009), including a closely related *P. protegens* strain, FD6, controlling *B. cinerea* on harvested tomato fruits (Zhang et al., 2020).

Notably, though deleting *phlA* improved the ability of *P. protegens* Pf-5 to inhibit *B. cinerea in vitro* (Figure 2), it nevertheless compromised the biocontrol activity of the bacterium *in planta* (Figure 3). While the reason for this discrepancy remains unclear, environmental differences between *in vitro* and *in planta* conditions may likely explain why a stronger antifungal effect of mutant ∆*phlA*/pUCP22 *in vitro* did not correlate with a better biocontrol protection *in planta*. Indeed, it is possible that the biosynthesis of bacterial antibiotics, their degradation and/or toxic effects toward the fungus, can be impacted by environmental factors in cannabis tissues, as previously suggested when comparing the results of *in vitro* cultures and of mushroom tissues inoculated with *P. protegens* Pf-5 (Henkels et al., 2014). The main disadvantage of *in vitro* confrontation assays is that secondary metabolite production can reach much higher amounts than in natural habitats, depending on the chosen nutritive medium which is often hundreds of times richer and allows ideal diffusion of the antibiotics through the agar (Köhl et al., 2019).While antibiotic detection *in planta* could provide useful information, the quantification of bacterial metabolites in natural substrates is notoriously difficult because of low recovery rates and production below limit of detection (Henkels et al., 2014), microbial degradation and chemical instability (Fernando et al., 2005), or interferences with the extraction and chromatography processes due to organic components in plant tissues (Rodriguez and Pfender, 1997).

Finally, corroborating the results of *P. protegens* FD6 deletion mutants against grey mold on tomato fruits (Zhang et al., 2020), the biocontrol activity of *P. protegens* Pf-5 deletion mutants against grey mold on cannabis leaves was significantly impaired but not completely abolished (Figure 3), suggesting that secondary metabolites other than PLT and DAPG also contribute to the antagonistic effect against *B. cinerea*. Indeed, other compounds widely produced by *Pseudomonas* strains also have deleterious effects on *B. cinerea* growth and development, like pyrrolnitrin (Janisiewicz and Roitman, 1988; Ajouz et al., 2011; Chang et al., 2011), hydrogen cyanide (Strano et al., 2017), rhizoxins (Loper et al., 2008) and phenazines (Schoonbeek et al., 2002; Zhang et al., 2015; Simionato et al., 2017). The efficient two-step mutagenesis method used here (Figure 1) could thus prove useful in future work to investigate the remaining contribution of some of these compounds in the observed biocontrol.

Likewise, the bacterial mutants obtained here could be exploited to investigate alternative modes of action of PLT and DAPG in plant protection. Indeed, in *A. thaliana*, DAPG-mediated induced systemic resistance (ISR) elicitation was shown to confer resistance against foliar pathogens *B. cinerea* and *Pseudomonas syringae* after priming with beneficial *Pseudomonas* strains Pf-5, Q2-87 and/or pure DAPG (Weller et al., 2012; Chae et al., 2020). However, DAPG production by another *Pseudomonas* strain, CHA0, did not elicit ISR against either of these two pathogens in *A. thaliana*, whereas it did against *Hyaloperonospora arabidopsidis* (formerly *Peronospora parasitica*) (Iavicoli et al., 2003), suggesting that ISR elicitation may depend on distinctive plant-microorganism interactions. In cannabis, so far, inoculations with non-DAPG producers *Pseudomonas simiae* WCS417 or *Pseudomonas synxantha* LBUM223 were unsuccessful at eliciting ISR against *B. cinerea* (Balthazar et al., 2020), hence DAPG producers like strain Pf-5 used in this study could be considered for future work.

Understanding the mode of action of biocontrol agents is essential to develop effective biocontrol products that pose no risk to humans or the environment (Köhl et al., 2019). In this regard, specific challenges and opportunities to consider when developing inoculants for cannabis crops can be found in a recent review dedicated to *Pseudomonas* spp. (Balthazar et al., 2022a). Notably, the risk for resistance development within the pathogen population seems to be of particular interest when considering antagonistic biocontrol agents acting through antibiosis. Indeed, raising concerns about the potential loss of efficacy of broadly used biocontrol agents like beneficial *Pseudomonas* spp., it has been reported that *B. cinerea* mutants can exhibit reduced sensitivity to the antibiotics phenazines (Schoonbeek et al., 2002), pyrrolnitrin (Ajouz et al., 2010, 2011; Fillinger et al., 2012) and DAPG (Schouten et al., 2008) under laboratory conditions. However, under field conditions, the risk of resistance build-up appears to be lower because small concentrations of antimicrobial compounds are produced by beneficial organisms interacting intermittently with the pathogen, especially if compared to large-scale applications of purified antimicrobial compounds produced by fermentation and applied at high doses to the entire crop (Köhl et al., 2019). Additionally, the use of biological control agents with multiple antimicrobial metabolites and/or modes of action, like beneficial *Pseudomonas* spp., may also help alleviate selection pressure and ensure lasting efficacy compared to isolated compounds (Ajouz et al., 2011). For example, in the case of DAPG, which does not target a specific protein as mode of action (Troppens et al., 2013), field isolates of the take-all pathogen *G. tritici* did not become more DAPG-resistant even after decades of wheat monoculture and exposure to populations of DAPG-producing *Pseudomonas* spp. (Kwak et al., 2009). Moreover, resistance to pyrrolnitrin in laboratory-induced *B. cinerea* mutants has been associated with a reduced fitness, suggesting that these mutants may not persist under natural conditions (Ajouz et al., 2010) and potentially explaining why they have not been found in fields so far (Fillinger et al., 2012). Altogether, these results are thus encouraging regarding the sustainable use of antibiotic-producing *Pseudomonas* spp. as biocontrol agents (Biessy and Filion, 2021).

### Conclusion and perspectives

In conclusion, this work suggests that the antibiotics PLT and DAPG are key determinants of *P. protegens* Pf-5 biocontrol success against the gray mold disease in cannabis. As *P. protegens* Pf-5 was previously demonstrated to inhibit a broad range of cannabis phytopathogens, these results might contribute to address the rising issue of emerging diseases causing severe yield and harvest quality losses in cannabis crops. In particular, screening for PLT-and DAPG-producing *Pseudomonas* strains seems to be indicated for the development of effective biocontrol products against devastating cannabis phytopathogens like *B. cinerea*. Moreover, this result might have further implications for the design of consortia combining such strains with other beneficial microorganisms, by dictating microbial compatibility (viability in combined formulations) and complementarity (offering different and/or synergistic modes of action). Future endeavors aimed at deciphering the molecular basis of pathogen susceptibility to PLT and DAPG, potential impacts on pathogen epidemiology and pathogenesis processes, as well as associated cannabis immune responses and microbiome changes after bacteria inoculations, should also provide useful avenues toward the development of effective biocontrol products.

## Data availability statement

The original contributions presented in the study are included in the article/Supplementary material, further inquiries can be directed to the corresponding author.

## Author contributions

CB, RS-O, GL, SL, DJ, and MF contributed to the conception and design of the study. RS-O generated the bacterial mutants and wrote the corresponding methods, Table 1, and all supplementary materials. GL and CB designed and performed the inhibition experiments *in vitro*. CB designed and performed the experiments *in planta*, analyzed experimental data, designed the main figures, and wrote the manuscript. SL, DJ, and MF supervised the project and contributed reagents, equipment, and/or funds. All authors contributed to the article and approved the submitted version.

## Funding

Translating Research into Innovation for Cannabis Health (TRICHUM) at Université de Moncton is supported by grants from Genome Canada (Genome Atlantic NB-RP3), the Atlantic Canada Opportunity Agency (project 212090), and the New Brunswick Innovation Foundation (RIF2018-036), Mitacs, and Organigram, Mitacs through the Mitacs Accelerate program.

## Conflict of interest

The authors declare that the research was conducted in the absence of any commercial or financial relationships that could be construed as a potential conflict of interest.

## Publisher’s note

All claims expressed in this article are solely those of the authors and do not necessarily represent those of their affiliated organizations, or those of the publisher, the editors and the reviewers. Any product that may be evaluated in this article, or claim that may be made by its manufacturer, is not guaranteed or endorsed by the publisher.
