# Supplementary material for: Pyoluteorin and 2,4-diacetylphloroglucinol are major contributors to Pseudomonas protegens Pf-5 biocontrol against Botrytis cinerea in cannabis
Source: Front Microbiol. 2022 Aug 9;13:945498. doi: 10.3389/fmicb.2022.945498 (PMC9395707; doi:10.3389/fmicb.2022.945498)
Supplement: Supplementary file 1 [file Data_Sheet_1.PDF]

SUPPLEMENTARY TABLE S1 | PCR primers.

Primer[a]	Sequence[b]	Relevant restriction site
**Cloning allelic exchange vectors**		
phlA-GA-UPF	**TAA AAC GAC GGC CAG TGC CA**A GCT TCC TTG TTC TCG AAG TGG TAG	
phlA-GA-UPR	**GGC GTA ATC A**CA CGT TCA TTT TCC TCT TG	
phlA-GA-DWNF	**AAT GAA CGT G**TG ATT ACG CCT GTC TAC GC	
phlA-GA-DWNR	**TAC GAA TTC GAG CTC GGT AC**C CAC GAT CAG TAC GTG GTT GC	
pltD-GA-UPF	**TAA AAC GAC GGC CAG TGC CA**A GCT TCT GTT CAC CGA ACT CAA GC	
pltD-GA-UPR	**TCT CCT TGC G**CA TAA CCA CCT CAA TCA CAG	
pltD-GA-DWNF	**GGT GGT TAT G**CG CAA GGA GAG TTG AAA TGG	
pltD-GA-DWNR	**TAC GAA TTC GAG CTC GGT AC**C ATA GCC CAG CAT GAC CGT AG	
**Cloning complementation vectors**		
phlA-ComplF	TGC ACT GGT ACC TGC TTC TGA GCA GGC ACG AC	*Kpn*I
phlA-ComplR	TGC ACT GGA TCC GCG TAG ACA GGC GTA ATC AG	*Bam*HI
pltD-ComplF	TGC ACT GGT ACC CTG CGT GGC CGA GGC CTG AC	*Kpn*I
pltD-ComplR	TGC ACT GGA TCC TCA GTC CAT TTC AAC TCT CCT TG	*Bam*HI
**Confirming strain genotypes**		
phlA-CKF	CAG GTT TCA TCA GGT GTG GC	
phlA-CKInt	ATA GGA TTC GGT CAG GTC GC	
phlA-CKR	CAT CTT GGC GTC CTT GAG TG	
pltD-CKF	GCG AGT TTC TTG AGT GTC CG	
pltD-CKInt	ATA GGG AAT CGT CGC CTC AC	
pltD-CKR	CGA GTG TTC ATT GCC ACA GG	

[a] Primers were purchased from Integrated DNA Technologies.

[b] Long 5′ extensions facilitating DNA assembly are bolded. Six-nucleotide 5′ extensions, included to increase the cleavage efficiency of restriction enzymes, are wavy-underlined. Restriction sites are underlined. Engineered stop codons are boxed.

SUPPLEMENTARY TABLE S2 | PCR amplification conditions.

Gene	PCR product[a]	Forward primer[b]	Reverse primer[b]	Product length (bp)	Annealing temperature (°C)	Extension time (min:sec)
**Cloning allelic exchange vectors[c]**						
phlA	Up	phlA-GA-UPF	phlA-GA-UPR	2,381	57.4	1:16
	Down	phlA-GA-DWNF	phlA-GA-DWNR	2,410	62.3	1:16
pltD	Up	pltD-GA-UPF	pltD-GA-UPR	2,368	59.0	1:16
	Down	pltD-GA-DWNF	pltD-GA-DWNR	2,287	62.1	1:16
**Cloning complementation vectors[c]**						
phlA	CDS	phlA-ComplF	phlA-ComplR	1,183	63.8	0:50
pltD	CDS	pltD-ComplF	pltD-ComplR	1,738	59.6	0:50
**Confirming strain genotypes[d]**						
phlA	Wild-type	phlA-CKF	phlA-CKInt	590	58.5	1:00
	Mutant	phlA-CKF	phlA-CKR	286	58.1	1:00
	Complement	phlA-ComplF	phlA-ComplR	1,183	57.0	1:00
pltD	Wild-type	pltD-CKF	pltD-CKInt	310	58.5	1:00
	Mutant	pltD-CKF	pltD-CKR	251	58.5	1:00
	Complement	pltD-ComplF	pltD-ComplR	1,738	53.7	1:00

[a] Up, upstream flank; Down, downstream flank; CDS, coding sequence.

[b] Primer sequences are provided in **Supplementary Table S1**.

[c] Flanking DNA and gene coding sequences were PCR-amplified using Phusion High-Fidelity DNA Polymerase (New England Biolabs). Each 50-µL reaction comprised 1 × Phusion GC Reaction Buffer (New England Biolabs), 200 µM each dNTP (Invitrogen), 5% v/v dimethyl sulfoxide (New England Biolabs or Thermo Scientific), 500 nM each primer (Integrated DNA Technologies), 4 ng μL^{-1} *P. protegens* Pf-5 genomic DNA, and 0.02 U μL^{-1} Phusion HF DNA Polymerase (New England Biolabs). The following PCR cycling conditions were used: (i) an initial 3-min denaturation at 98°C, (ii) 35 amplification cycles comprising a 10-sec denaturation at 98°C, a 30-sec annealing at an optimized temperature, and an extension at 72°C, and (iii) a final 5-min extension at 72°C.

[d] The wild-type/mutant loci (allelic exchange vectors, deletion mutants) and the coding sequences (complementation vectors) were PCR-amplified using DreamTaq Hot Start Green PCR Master Mix (Thermo Scientific). Each 20-µL reaction comprised 1 × DreamTaq Hot Start Green PCR Master Mix, 5% v/v dimethyl sulfoxide, 300 nM each primer, and either crude cell lysate or 2.5 ng μL^{-1} plasmid/genomic DNA as template. The following PCR cycling conditions were used: (i) an initial 3-min denaturation at 95°C, (ii) 35 amplification cycles comprising a 30-sec denaturation at 95°C, a 30-sec annealing at an optimized temperature, and an extension at 72°C, and (iii) a final 5-min extension at 72°C.

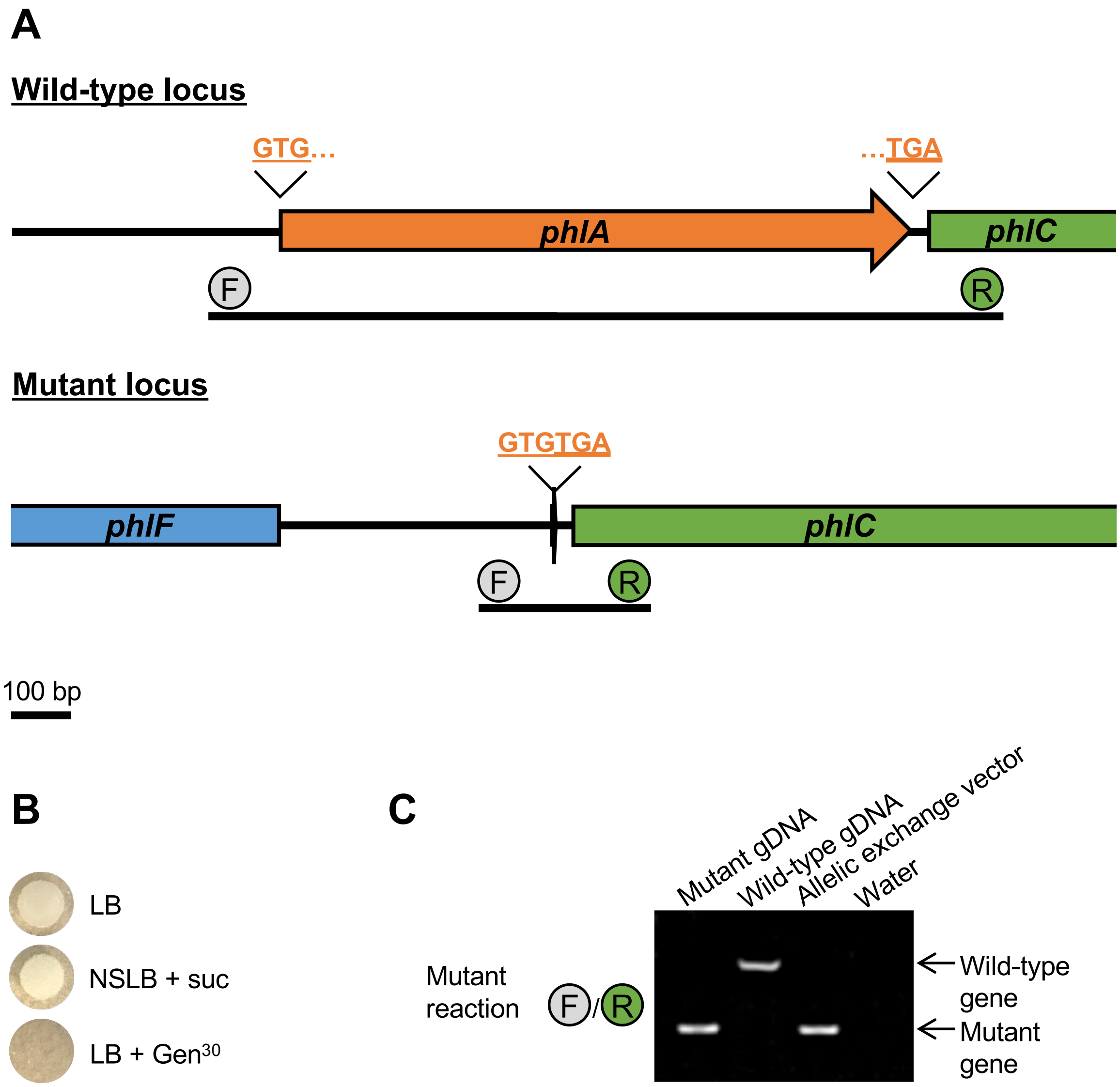

SUPPLEMENTARY FIGURE S1 | In-frame deletion of the *phlA* gene in *P. protegens* strain Pf-5. **(A)** Schematic representation of the *phlA* locus in the wild-type strain Pf-5 and the deletion mutant strain Pf-5Δ*phlA* (**Table 1**). Block arrows represent coding regions. Native start and stop codons are underlined once and twice, respectively. Solid black lines specify the regions amplified during diagnostic PCRs. Schematics were drawn to scale. **(B)** Loss of the allelic exchange vector backbone was confirmed using phenotypic assays. The deletion mutant strain Pf-5Δ*phlA* was grown at 25°C for 24 h on 30 mL Lennox's LB agar (LB), no-salt LB agar supplemented with 10% w/v sucrose (NSLB + suc), and Lennox's LB agar supplemented with 30 $\mu g\ mL^{-1}$ gentamicin sulfate (LB + Gen^{30}). **(C)** The genotype of the deletion mutant was confirmed by PCR-amplifying the *phlA* locus from genomic DNA isolated from the deletion mutant strain Pf-5Δ*phlA* (Mutant gDNA) using primers phlA-CKF (F) and phlA-CKR (R) (**Supplementary Tables S1 and S2**). Genomic DNA extracted from the wild-type strain Pf-5 (Wild-type gDNA) and plasmid pEX18Gm-Δ*phlA* (Allelic exchange vector) served as controls. Water, no-template negative control.

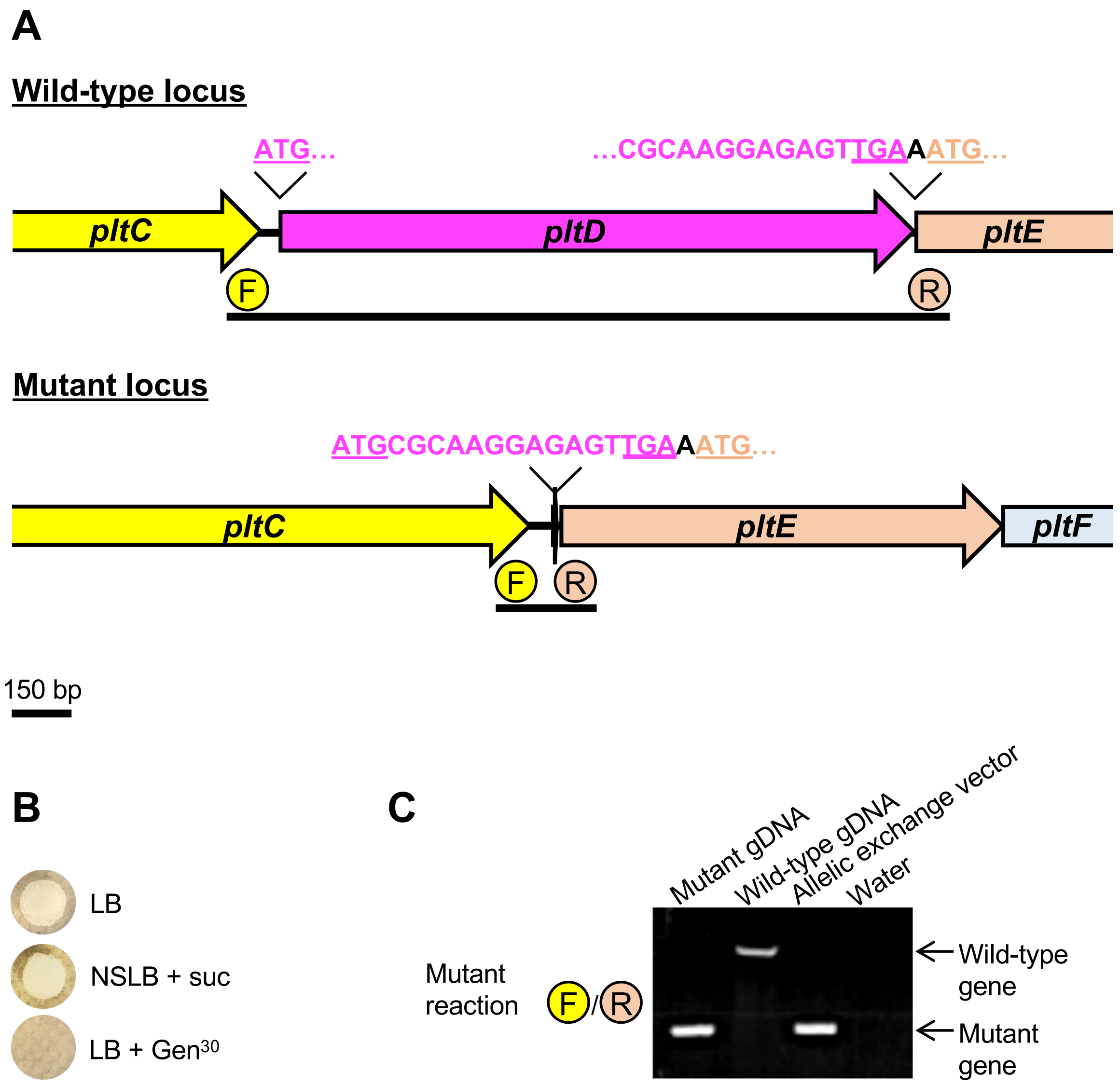

SUPPLEMENTARY FIGURE S2 | In-frame deletion of the *pltD* gene in *P. protegens* strain Pf-5. **(A)** Schematic representation of the *pltD* locus in the wild-type strain Pf-5 and the deletion mutant strain Pf-5Δ*pltD* (**Table 1**). Block arrows represent coding regions. Native start and stop codons are underlined once and twice, respectively. Solid black lines specify the regions amplified during diagnostic PCRs. Schematics were drawn to scale. **(B)** Loss of the allelic exchange vector backbone was confirmed using phenotypic assays. The deletion mutant strain Pf-5Δ*pltD* was grown at 25°C for 24 h on 30 mL Lennox's LB agar (LB), no-salt LB agar supplemented with 10% w/v sucrose (NSLB + suc), and Lennox's LB agar supplemented with 30 μg mL^{-1} gentamicin sulfate (LB + Gen^{30}). **(C)** The genotype of the deletion mutant was confirmed by PCR-amplifying the *pltD* locus from genomic DNA isolated from the deletion mutant strain Pf-5Δ*pltD* (Mutant gDNA) using primers pltD-CKF (F) and pltD-CKR (R) (**Supplementary Tables S1 and S2**). Genomic DNA extracted from the wild-type strain Pf-5 (Wild-type gDNA) and plasmid pEX18Gm-Δ*pltD* (Allelic exchange vector) served as controls. Water, no-template negative control.

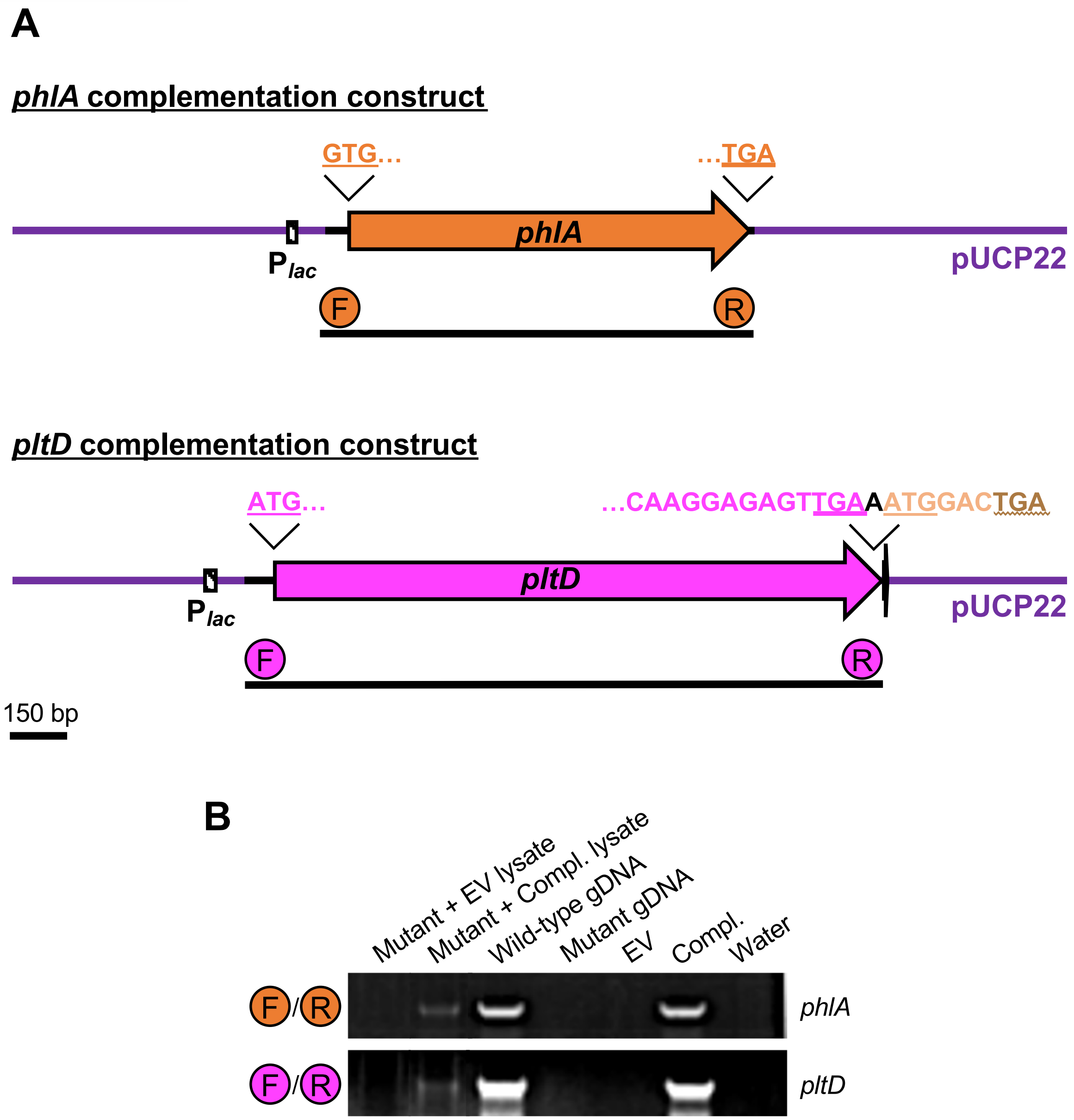

SUPPLEMENTARY FIGURE S3 | Complementation of *P. protegens* deletion mutants Pf-5Δ*phlA* and Pf-5Δ*pltD*. (A) Schematic representation of the *phlA* and *pltD* loci in the complementation plasmids pUCP22-*phlA* and pUCP22-*pltD*, respectively (**Table 1**). Block arrows and hatched boxes represent coding regions and promoters, respectively. Native start and stop codons are underlined once and twice, respectively, and engineered stop codons are underlined with a wavy line. Solid black lines specify the regions amplified during diagnostic PCRs. Schematics were drawn to scale. **(B)** The genotype of the complementation mutants was confirmed by PCR-amplifying the *phlA* or *pltD* gene using the appropriate ComplF (F) and ComplR (R) primers (**Supplementary Tables S1 and S2**). Crude lysate of the complemented mutant strain Pf-5Δ*phlA*/pUCP22-*phlA* or Pf-5Δ*pltD*/pUCP22-*pltD* (Mutant + Compl. lysate), respectively, served as template. The following controls were included: crude lysate of the mutant strain carrying the empty vector pUCP22 (Mutant + EV lysate), genomic DNA extracted from the wild-type strain Pf-5 (Wild-type gDNA), genomic DNA isolated from the mutant strain Pf-5Δ*phlA* or Pf-5Δ*pltD* (Mutant gDNA), the empty vector pUCP22 (EV), the complementation plasmid pUCP22-*phlA* or pUCP22-*pltD* (Compl.), and water (no-template negative control).
